# Central nervous system injury meets nanoceria: opportunities and challenges

**DOI:** 10.1093/rb/rbac037

**Published:** 2022-06-02

**Authors:** Wang Yang, Maoting Zhang, Jian He, Mingfu Gong, Jian Sun, Xiaochao Yang

**Affiliations:** School of Biomedical Engineering and Medical Imaging, Army Medical University, Chongqing 400038, China; Army Health Service Training Base, Army Medical University, Chongqing 400038, China; School of Biomedical Engineering and Medical Imaging, Army Medical University, Chongqing 400038, China; School of Biomedical Engineering and Medical Imaging, Army Medical University, Chongqing 400038, China; Xinqiao Hospital, Army Medical University, Chongqing 400038, China; School of Biomedical Engineering and Medical Imaging, Army Medical University, Chongqing 400038, China; School of Biomedical Engineering and Medical Imaging, Army Medical University, Chongqing 400038, China

**Keywords:** nanoceria, reactive oxygen species, central nervous system injury, stroke, neurotrauma

## Abstract

Central nervous system (CNS) injury, induced by ischemic/hemorrhagic or traumatic damage, is one of the most common causes of death and long-term disability worldwide. Reactive oxygen and nitrogen species (RONS) resulting in oxidative/nitrosative stress play a critical role in the pathological cascade of molecular events after CNS injury. Therefore, by targeting RONS, antioxidant therapies have been intensively explored in previous studies. However, traditional antioxidants have achieved limited success thus far, and the development of new antioxidants to achieve highly effective RONS modulation in CNS injury still remains a great challenge. With the rapid development of nanotechnology, novel nanomaterials provided promising opportunities to address this challenge. Within these, nanoceria has gained much attention due to its regenerative and excellent RONS elimination capability. To promote its practical application, it is important to know what has been done and what has yet to be done. This review aims to present the opportunities and challenges of nanoceria in treating CNS injury. The physicochemical properties of nanoceria and its interaction with RONS are described. The applications of nanoceria for stroke and neurotrauma treatment are summarized. The possible directions for future application of nanoceria in CNS injury treatment are proposed.

## Graphical Abstract

**Figure:**
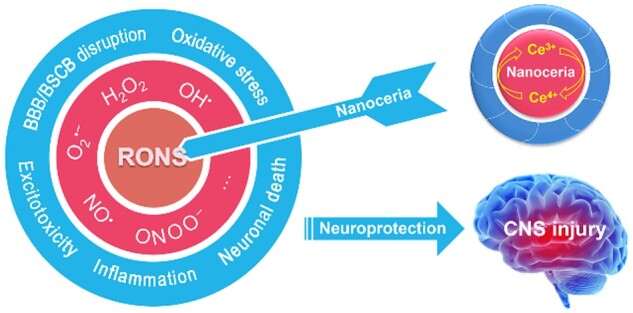

## Introduction

**Figure 1.**
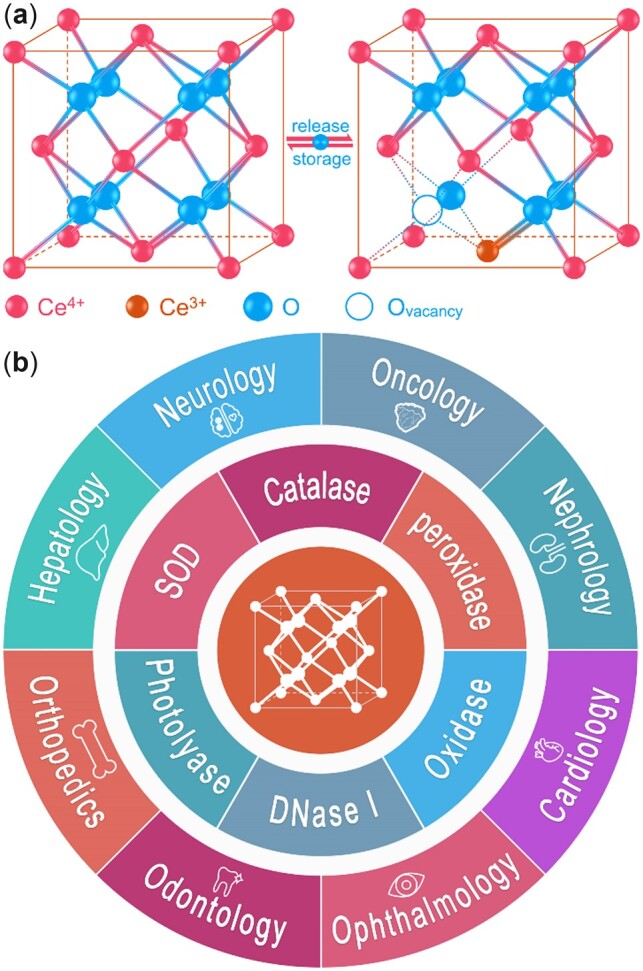
(**a**) Nanoceria owns a fluorite structure and can change between two valence states, accompanied by the formation of the oxygen vacancies. (**b**) Enzyme mimetic activities and biomedical applications of nanoceria.

As a well-known catalytic metal oxide, ceria has been widely and successfully utilized in industrial catalysis, especially for the fabrication of three-way catalytic converters for vehicle exhaust gas control [1–3]. Cerium has a specific [Xe]4f^1^5d^1^6s^2^ electron configuration, where the energy of the inner 4f level is nearly equal to that of the 6s level, allowing electrons to occupy these valence subshells variably. Due to this property, cerium ions can easily and reversibly switch between valence states of Ce^3+^ and Ce^4+^. Therefore, ceria is a crystal mixture of CeO_2_ and Ce_2_O_3_, and could partially exchange between CeO_2_ and Ce_2_O_3_ with structural integrity. During the Ce^4+^/Ce^3+^ transition, the electrons from oxygen ions localize in the 4f subshell of Ce^3+^, accompanied by the formation of oxygen vacancies (Fig. 1a) [4, 5], rendering ceria an excellent oxygen storage capacity [6]. Moreover, when ceria particle size decreases to nanoscale, the oxygen vacancy level and Ce^3+^/Ce^4+^ ratio get a sharp increase in the crystal structure. Correspondingly, the catalytic activities of nanoceria were significantly enhanced [7, 8]. Up to now, the engineered nanoceria can be prepared by diverse synthetic processes, such as precipitation [9, 10], hydrothermal [11], microemulsion [12], green synthesis [13], sonochemical and microwave-assisted [14] methods. Accordingly, nanoceria with different sizes, shapes and catalytic activities have been produced. More comprehensive reviews on the synthesis and properties of nanoceria were published [15]. Inspired by their intriguing physicochemical properties and catalytic performances, many studies have been conducted to explore the biological effects of nanoceria. Notably, nanoceria exhibits multiple enzyme-mimetic activities (Fig. 1b), including superoxide dismutase (SOD) [16], catalase [17], peroxidase [18], oxidase [19], DNase I [20] and photolyase [21] like activities. Benefited from these activities, nanoceria has shown great potential for biomedical applications in neurology [22–24], oncology [25–27], nephrology [28–30], hepatology [31–33], cardiology [34], ophthalmology [35–37], odontology [38–40], orthopedics [41–43] and others.

In the field of neurological diseases, central nervous system (CNS) injury (including stroke and neurotrauma) is associated with high morbidity and mortality worldwide [44–46]. Reactive oxygen and nitrogen species (RONS) play an important role in the pathological cascade of molecular events after CNS damage [47]. Because the human brain is the most oxygen supply-dependent organ [48, 49], consuming nearly 20% of the oxygen provided by the vasculature while accounting for only ∼2% of the body’s weight [50], the oxygen consumption disorder may significantly increase the RONS level in the CNS. Therefore, treatment with antioxidants can be a promising strategy against oxidative/nitrosative stress induced by RONS after CNS injury [51–53]. However, current antioxidants (e.g. vitamins, lipoic acids, polyphenols and carotenoids) utilized in RONS-related disease treatment have obtained limited therapeutic effects due to the innate drawback that these organic drugs can participate in only one redox cycle after which they inactivate, i.e. a single dose is quickly depleted *in vivo* and repetitive doses are still inadequate to control oxidative/nitrosative stress in many cases. Thus, more effective strategies in modulating the balance of RONS need to be developed. In the last few decades, nanotechnology has opened a new chapter in regenerative medicine and a variety of nanosized biomaterials with RONS regulating ability are springing up [54, 55]. Of these, some nanozymes possessing antioxidant activity including platinum [56], manganese [57], fullerene [58], melanin [59] and ceria [23] have shown neuroprotective effects in CNS disorders. Featured with many superiorities such as low cost, low toxicity and simple synthetic procedures, nanoceria is the most extensively explored RONS modulating nanozyme for CNS injury treatment. In addition, it is one of the first nanoparticles utilized as therapeutic agent [60, 61] and even the first material tested as an antioxidant in the space [62]. In this context, nanoceria is selected here as a representative antioxidant nanoparticle. With regenerative ability, nanoceria has an obvious advantage over the other antioxidants and may be a novel option against oxidative/nitrosative stress in the future. Hereinafter, this review discusses the RONS modulating ability of nanoceria, presents up-to-date advances in stroke and neurotrauma treatment and provides some future directions to bridge the gap between experiments and the clinic.

## Nanoceria as a RONS modulator

RONS are implicated in many physiological and pathologic processes of aging and disease. They are widely formed particularly through the electron transport chains in mitochondria, including free radicals with unpaired electrons (e.g. superoxide anion ($O_{2}^{•-}$), hydroxyl radical (OH^•^)) and some biologically important non-radicals (e.g. hydrogen peroxide (H_2_O_2_), peroxynitrite (ONOO^–^)) [63]. These species are essential for regulating internal homeostasis in living systems. However, excessive RONS production can induce oxidative/nitrosative stress, leading to important biomolecules (DNA, protein, lipids) damage and cell death, which is always involved in the early onset of disease [64].

The RONS neutralizing capacities of nanoceria have been discovered for only a dozen years. At the beginning of this century, Rzigalinski *et al.* [61, 65] serendipitously observed that nanoceria could prolong the lifespan of neuronal cells during their pioneering works, where nanoceria might function as a nanozyme or antioxidant modulating RONS. Since then, the enzyme-like activities and biomedical applications of nanoceria have gained extensive interest in the scientific literature. $O_{2}^{•-}$ is the precursor of most RONS and can be scavenged by SOD, then the SOD mimetic activity of nanoceria has been confirmed. The reaction mechanism follows by Equations (1) and (2) [16]. Further, there was a close correlation between Ce^3+^/Ce^4+^ ratio and SOD mimetic efficiency, while nanoceria with higher Ce^3+^/Ce^4+^ ratio was more effective than lower ratio ones for $O_{2}^{•-}$ clearance [16, 66].

(1) $$O_{2}^{\cdot -}+{\text{Ce}}^{4+}\to {\;O}_{2}+{\;\text{Ce}}^{3+}$$

(2) $$O_{2}^{\cdot -}+{\text{Ce}}^{3+}+2H^{+}\to {{\;H}_{2}O}_{2}+{\text{Ce}}^{4+}$$

During the dismutation process of $O_{2}^{•-}$, H_2_O_2_ is generated and can be degraded by catalase to water and oxygen in aerobic organisms. Soon after the discovery of SOD mimetic activity, nanoceria has also found catalase mimetic activity [17], under which the mechanism follows by Equations (3) and (4) [67]. It is worth noting that the decomposition of H_2_O_2_ accelerated by nanoceria is also correlated with the Ce^3+^/Ce^4+^ ratio but contrary to the SOD mimetic activity. For example, nanoceria with a lower Ce^3+^/Ce^4+^ ratio (7%) was more effective in H_2_O_2_ decomposition than those with a higher Ce^3+^/Ce^4+^ ratio (28%) [17]. Nevertheless, in a study comparing the catalytic activity of three different nanostructures (nanorods, nanocubes and nanooctahedra, with Ce^3+^ concentrations of 18.3%, 17.6% and 15.5%, respectively), the decomposition of H_2_O_2_ was positively correlated to the Ce^3+^/Ce^4+^ ratio of nanoceria [68]. These results indicate that in addition to Ce^3+^ concentration, the catalase mimetic activity of nanoceria may be influenced by other factors, e.g. shape and size.

(3) $${H_{2}O}_{2}+\text{Ce}\left(\text{III}\right)–\text{Ce}O_{2}\;\to {\;H}_{2}O+O=\text{Ce}\left(\text{IV}\right)$$

(4) $${H_{2}O}_{2}+O=\text{Ce}\left(\text{IV}\right)–\text{Ce}O_{2}\;\to \;H_{2}O+O_{2}+\text{Ce}\left(\text{III}\right)–\text{Ce}O_{2}$$

Because $O_{2}^{•-}$ and H_2_O_2_ are two dominant RONS implicated in disease processes, SOD and catalase are indispensable in the construction of the first line of defense against oxidative stress [69]. *In vivo*, the combination of NO^•^ and $O_{2}^{•-}$ leads to the formation of ONOO^–^, a highly reactive molecule that can induce enzyme inactivation, DNA damage and lipid peroxidation [70]. Hence, the interaction between ONOO^–^ and nanoceria has been tested. As a result, nanoceria significantly promoted the decay of this destructive molecule, while the decay rate seemed irrelevant to the Ce^3+^/Ce^4+^ ratio according to the experimental data [71]. In addition, it was reported that nanoceria could scavenge NO^•^ radicals directly or indirectly, which might contribute to less production of ONOO^–^ [72].

Hydroxyl radical (OH^•^), one of the strongest RONS that must be mentioned, is generated primarily by the Fenton reaction *in vivo*. Nanoceria acting as OH^•^ scavenger has been demonstrated by establishing a photometric system *in vitro*. The OH^•^ clearance activity was size-dependent and closely correlated with Ce^3+^ ions at the surface of the nanoparticles, while the smaller nanoceria (size, 5–10 nm; Ce^3+^/Ce^4+^ ratio, 30%) captured more OH^•^ than the larger (size, 15–20 nm; Ce^3+^/Ce^4+^ ratio, 20%) [73]. It was shown as well that nanoceria could inactivate stable nitroxyl radical, and the inactivation rate increased with the particle size decreasing [74].

**Figure 2.**
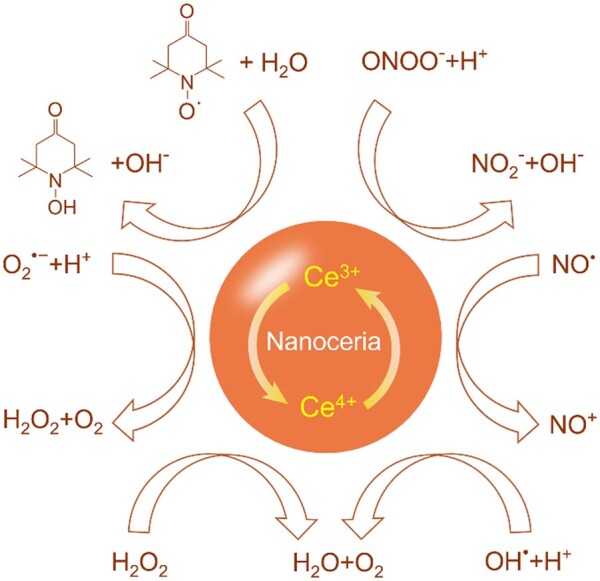
The main types of RONS can be modulated by nanoceria via an autocatalytic mechanism of reversible Ce^4+^/Ce^3+^ transition.

Generally, nanoceria can modulate multiple RONS types via an autocatalytic mechanism of reversible Ce^4+^/Ce^3+^ transition (Fig. 2). Thus, a single dose of nanoceria can maintain long-lasting antioxidant effect in biological systems. For example, in an *in vitro* ischemia model of hippocampal injury, 5.8 µM nanoceria achieved the same neuroprotective effects as 10 mM (nearly 1700-fold concentration of nanoceria) n-acetylcysteine, a benchmarked antioxidant commonly used to evaluate the activity of other antioxidants [75, 76]. Notably, the mechanism of RONS clearance is different between nanoceria and traditional organic antioxidants, since the former directly acts as an oxygen receptor while the latter is actually a hydrogen donor. In addition, the organic antioxidants scavenge RONS by single electron exchange with these species and in turn transform themselves into radicals acting as ‘prooxidants’. Therefore, nanoceria is a more efficient antioxidant for oxidative stress.

## Nanoceria in CNS injury treatment: targeting RONS

**Figure 3.**
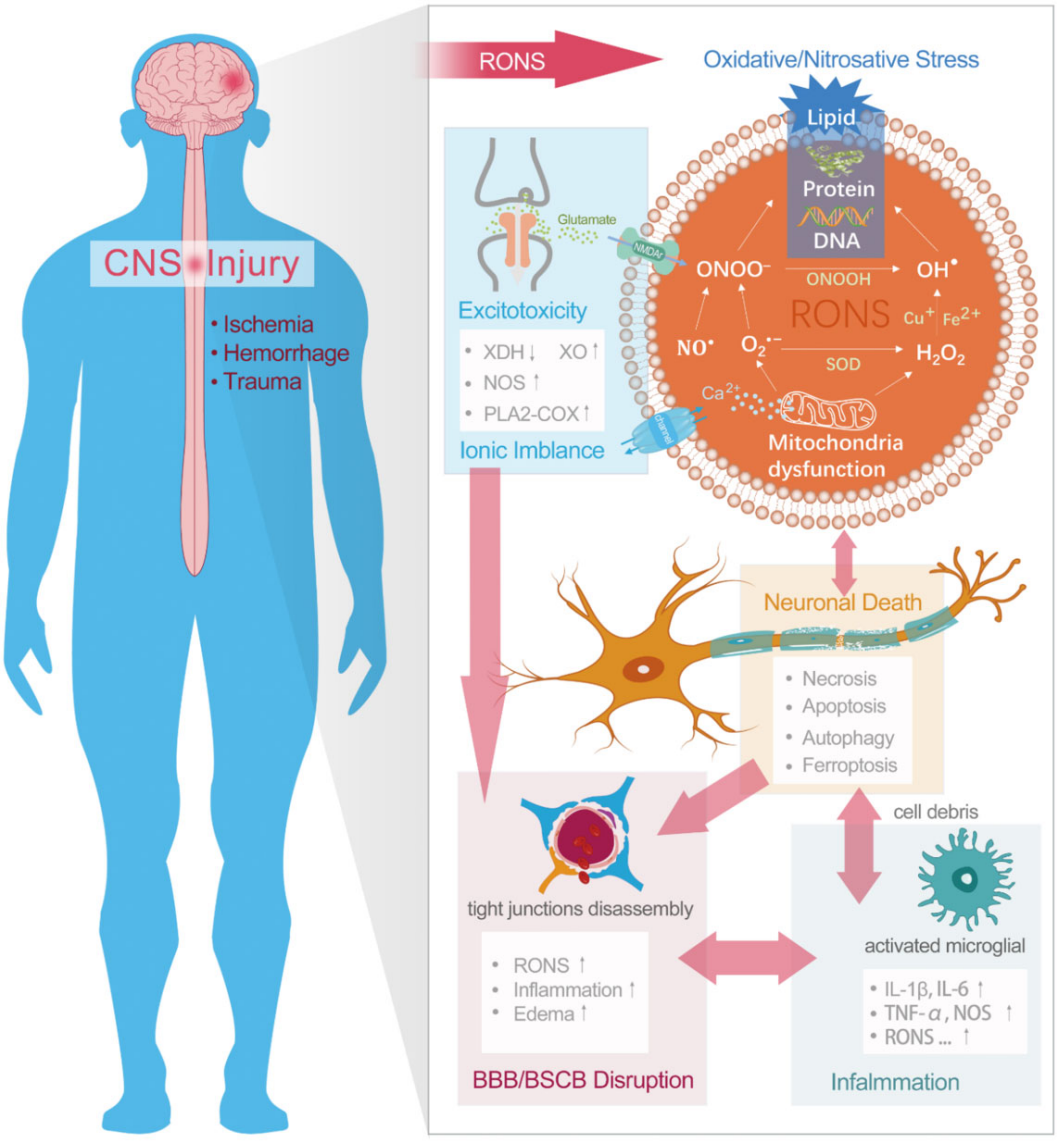
The major pathways involved in CNS injury: oxidative/nitrosative stress, excitotoxicity, ionic imbalance, inflammation, BBB/BSCB disruption and neuronal death. There is extensive crosstalk among these pathways, in which RONS play a critical role. XDH, xanthine dehydrogenase; XO, xanthine oxidase; NOS, nitric oxide synthase; PLA2, phospholipase A2; COX, cyclooxygenase; NMDAr, *N*-methyl-d-aspartic acid receptor; IL-1β, interleukin-1β; IL-6, interleukin-6; TNF-α, tumor necrosis factor α.

The most challenging and leading types of CNS injury are stroke and neurotrauma. Stroke, a brain attack, is induced by either blockage (ischemic stroke) or leakage (hemorrhagic stroke) of blood vessels carrying oxygen and nutrients to the brain [77]. Traumatic injury to the brain or spinal cord shares many similar pathological features with stroke, including ischemia, hemorrhage, blood–brain barrier (BBB) or blood–spinal cord barrier (BSCB) disruption, neuronal death and inflammation. When stroke or neurotrauma occurs, part of the CNS may suffer from ischemia and hypoxia, followed by mitochondrial dysfunction and oxidative/nitrosative stress, leading to further neurological disorders. Additionally, during the injury progression, many other pathways (e.g. excitotoxicity, BBB/BSCB disruption, inflammation, neuronal death) are involved, and extensive crosstalk among these pernicious pathways dramatically increases as shown in Fig. 3 [47, 77]. In this crosstalk, excessive RONS play a pivotal and important role in triggering cell death (e.g. necrosis [78, 79], apoptosis [80], autophagy [81, 82], ferroptosis [83]) and delayed neurological deficits. With excellent RONS modulating ability, nanoceria has been utilized in various CNS-related diseases, ranging from Alzheimer’s disease [24, 84–87], Parkinson’s disease [22, 88], multiple sclerosis [89, 90], amyotrophic lateral sclerosis [76], and have we focused on stroke and neurotrauma [23, 91–105].

### Ischemic stroke

**Figure 4.**
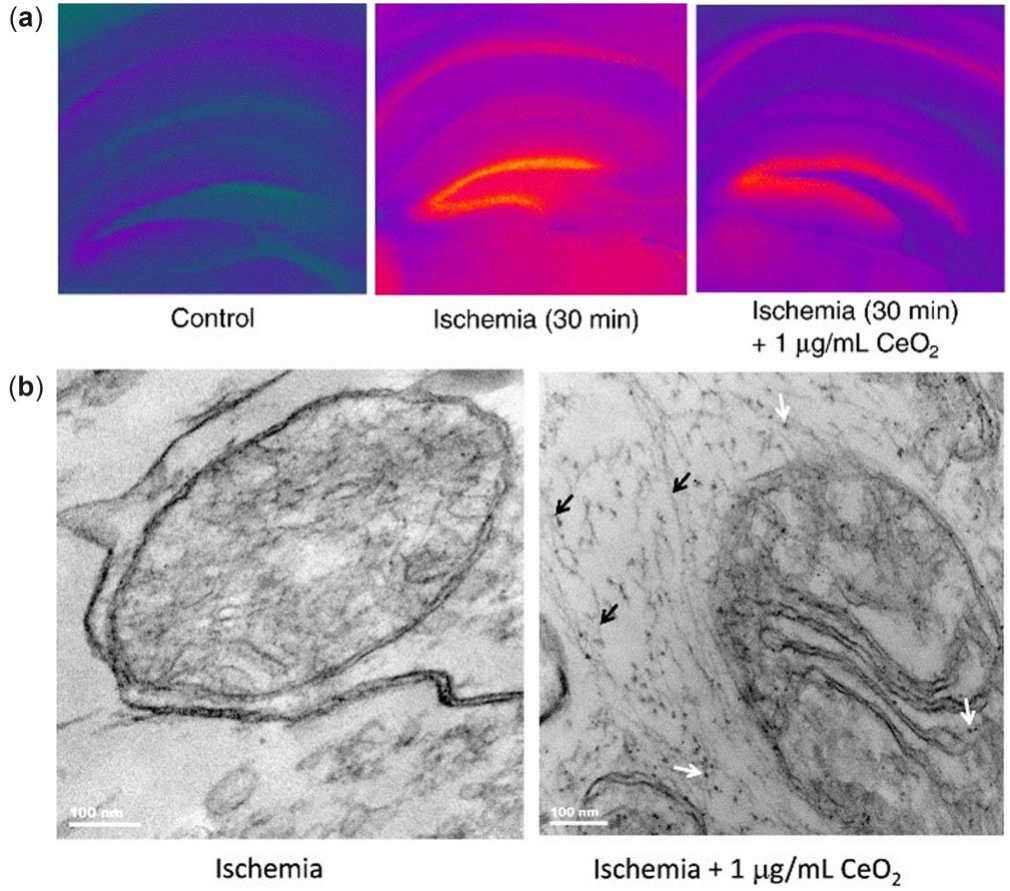
Neuroprotective effects of nanoceria in ischemia treatment. (**a**) The cell death of ischemic brain slice was significantly decreased by nanoceria. (**b**) T EM (transmission electron microscopy) micrographs of hippocampal brain slices 24 h post-ischemia. Nanoceria were located in the mitochondria and associated with neurofilaments. Adapted with permission from Ref. [75], Elsevier 2011.

Ischemic stroke is the most common subtype of stroke, accounting for more than 80% of all strokes [106, 107]. In a mouse hippocampal brain slice model of ischemia, many nanoparticles localized to the mitochondria, in which the mitochondrial cristae were highly organized after treatment with commercially available nanoceria (Fig. 4). Besides, a significant reduction (50%) in ischemic cell death was observed with a modest reduction (15%) in $O_{2}^{•-}$ and NO^•^. Interestingly, 3-nitrotyrosine induced by ONOO^–^ displayed a remarkable reduction (70%), which was far greater than that of $O_{2}^{•-}$ and NO^•^. Considering that both $O_{2}^{•-}$ and NO^•^ are precursors of ONOO^–^, their reductions may contribute to a significant decrease in ONOO^–^. These results imply that direct or indirect clearance of ONOO^–^ is an important mechanism underlying the neuroprotective effects of nanoceria after ischemia. Moreover, it is worth mentioning that the optimal dose of nanoceria in the study is 1 µg/ml, while a higher concentration (2 µg/ml) leads to agglomeration in artificial cerebrospinal fluid (CSF), which should be considered *in vivo* [75]. The agglomeration is a common phenomenon for nanomaterials under physiological conditions, especially in the bloodstream. When the particle size is smaller, the surface-to-volume ratio is larger, and nanoparticles are more likely to agglomerate and adsorb plasma proteins. Then, rapid clearance by macrophages can happen before nanoceria is transported to target cells [108]. In the case of stroke, small particles (<5 nm) are more favorable to cross the BBB into the brain [109, 110]. This dilemma is a major barrier to the design of novel nanomedicine in brain disease treatment. One possible approach for nanoceria overcoming this problem is surface modification to decrease agglomeration and protein adsorption, and simultaneously increase the circulation time in the blood [111]. Recently, ∼2.5-nm nanoceria modified with different ratios of citrate acid (CA) and ethylenediamine tetraacetic acid (EDTA) was applied in the brain slice model. As a result, the antioxidant activity of nanoceria was influenced by the surface coatings and nanoparticles with a 1:1 ratio of CA/EDTA exerted a stronger neuroprotective performance. After being injected into a rat model of ischemia–reperfusion 72 h in advance, the CA/EDTA-coated nanoceria achieved a 52% reduction of superoxide accumulation in the hippocampus [94].

The first case of nanoceria protecting against stroke *in vivo* has been reported by Kim *et al.* In this work, uniform 3-nm nanoceria with phospholipid-PEG modification was well dispersed in both phosphate-buffered saline (PBS) and plasma, exhibiting dose-dependent SOD/catalase mimetic activities. After the ischemia–reperfusion rat received a 0.5 mg/kg intravenous injection of nanoceria, the brain infarct area was considerably reduced by 50%. Additionally, the phospholipid-PEG-capped nanoparticles accumulated in the ischemic hemisphere were far more abundant than those in the contralateral hemisphere, suggesting BBB disruption in the damaged area which promoted the entrance of nanoceria into the brain [23]. The higher accumulation of nanoceria in the brain is at least partly due to PEGylation and small particle size, which ameliorate its dispersion and prolong the circulation time in the vasculature. As PEG possesses uncharged hydrophilic residues and high surface mobility, the biocompatibility of PEGylated nanoceria in living systems is significantly improved [112] while the radical scavenging ability is little reduced compared to bare nanoceria [113]. Although BBB disruption after stroke facilitates nanoparticle permeation into the damaged area of the brain, the passage rate is still very low compared to the total injection dosage. Consequently, the therapeutic effect is quite limited, and a huge portion of nanoparticles may be transported to other organs, possibly inducing side effects.

**Figure 5.**
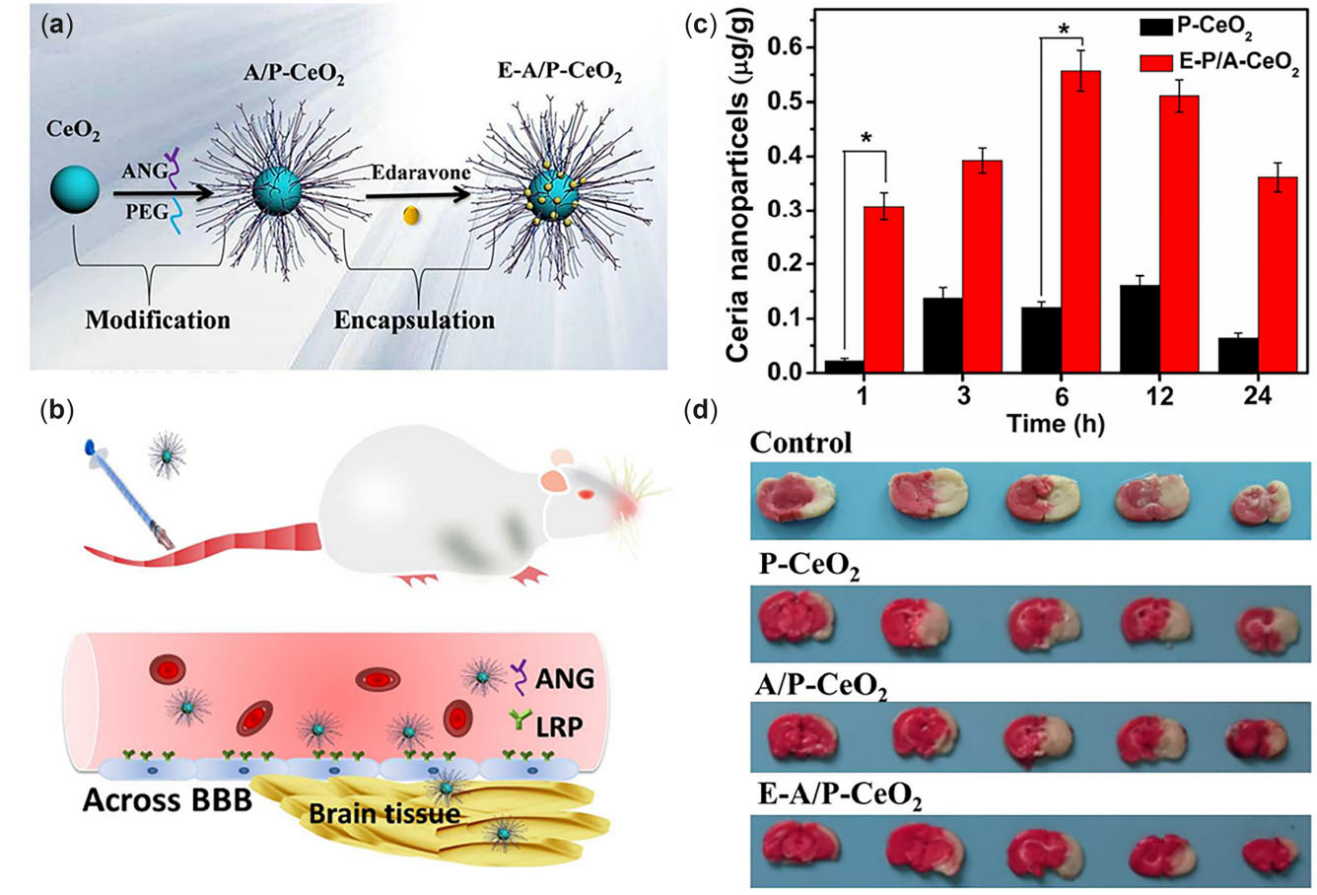
Schematic illustration of (**a**) the synthetic procedure of E-a/P-CeO_2_ and (**b**) the ANG-targeting to the overexpressed LRP on BCECs, (**c**) which facilitated the nanoparticles to penetrate BBB into brain tissue and thus (**d**) reduced the infarct volume of the ischemic brain most effectively. Adapted with permission from Ref. [91], © American Chemical Society 2018.

More importantly, the breakdown of the BBB may allow hazardous molecules and compounds to enter the brain as well, resulting in additional BBB damage and neurological dysfunction. Concerning this issue, functionalized core-shell nanoceria coated with angiopep-2/PEG and loaded with edaravone have achieved simultaneous intracerebral uptake and BBB protection in stroke treatment (Fig. 5). There are three key takeaway points in the nanocomposite system: (i) PEGylation improves biocompatibility, (ii) angiopep-2 targets the BBB and (iii) nanoceria and edaravone scavenge RONS [91]. Angiopep-2 is a specific ligand that can be recognized by low-density lipoprotein receptor-related protein (LRP) overexpressed on cells at the BBB, which facilitates the passage of nanoceria into brain tissue via a transcytosis process [114]. PEGylated nanoceria with angiopep-2 modification obtained much higher accumulation in the brain tissue of healthy rats after injection indicating that angiopep-2 played a critical role in BBB targeting and crossing. Moreover, when loaded with edaravone (a clinical drug applied in stroke treatment), angiopep-2/PEGylated nanoceria exhibited the most protective effects on BBB integrity. The synergetic clearance of RONS by nanoceria and edaravone could alleviate cell death and disassembly of the tight junctions [91].

**Figure 6.**
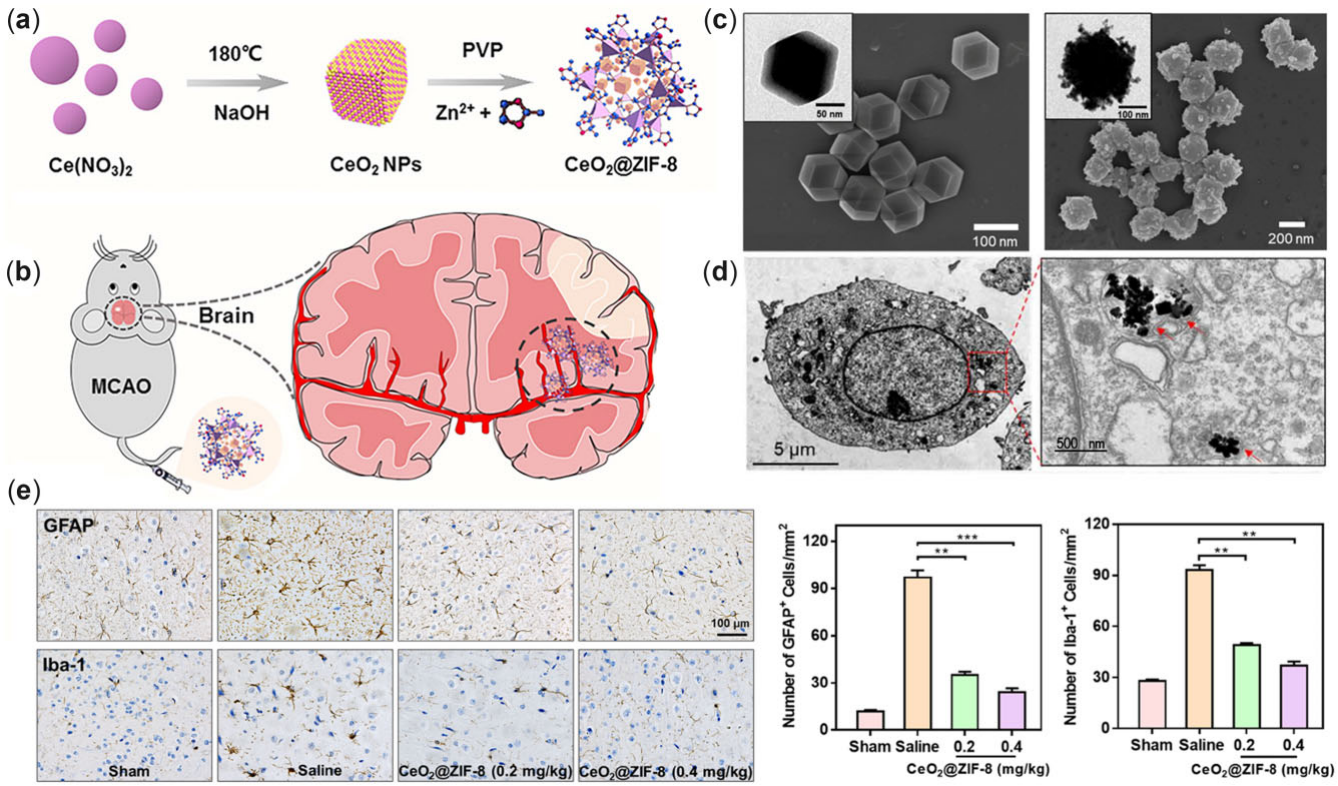
(**a**) Schematic illustration of CeO_2_@ZIF-8 synthesis and (**b**) its application in ischemic stroke treatment. (**c**) TEM and SEM (scanning electron microscope) images of ZIF-8 (left) and CeO_2_@ZIF-8 (right) nanocomposites. (**d**) TEM image of CeO_2_@ZIF-8 internalized in PC12 cells. The CeO_2_ nanopolyhedra was found in the lysosome but the outer ZIF-8 framework mostly degraded in the acidic lysosomal environment. © Treatment with CeO_2_@ZIF-8 significantly down-regulated the expression of GFAP (glial fibrillary acidic protein; a marker of astrocytes) and iba-1 (ionized calcium-binding adaptor molecule-1; a marker of microglia) in ischemic brain sections. Adapted with permission from Ref. [95], CC by-NC 4.0, © The Author(s) 2020.

Notably, ischemic stroke can promote BBB leakage and active angiogenesis of microvessels in the penumbra [115], during which vascular endothelial growth factor (VEGF) is known to play an important role [116]. Interestingly, nanoceria with high surface area and Ce^3+^/Ce^4+^ ratio led to robust angiogenesis by modulating oxygen in the intracellular environment [117]. In a study by our group, it was observed that nanoceria enhanced vascularization by triggering high expression of the angiogenic factor VEGF [41], which could shed light on the leakage and angiogenesis of the BBB. In other experiments, nanoceria showed benefits in alleviating BBB disruption after ischemia [92, 93]. By targeting integrin αvβ3 (a cell-surface receptor protein selectively upregulated after ischemia), custom synthesized biotinylated-LXW7-nanoceria could effectively arrive at the ischemic penumbra region and prevent brain damage [92]. LXW7, incorporated into this nanocomposite system, not only facilitates nanoparticles targeting damaged areas but also inhibits integrin αvβ3, preserving the BBB in the early stage [118]. In addition to the BBB, oxidative/nitrosative stress during stroke can damage endothelial cells, inducing vascular lesions and additional cerebral hemorrhage. Nanoceria coated with phosphonic acid PEG copolymers exhibited protective effects by reducing glutamate-induced production of RONS in cerebral endothelial cells [119]. More recently, following an *in situ* synthetic method, nanoceria was capped with ZIF-8, a porous metal-organic framework comprised imidazolate linkers and zinc ions (Fig. 6). Through a lysosome-mediated endocytic pathway, CeO_2_@ZIF-8 could be delivered into cells, after which the outer ZIF-8 shell decomposed and the inner core of CeO_2_ was released in the acidic lysosomal environment. The CeO_2_@ZIF-8 nanocomposite gained enhanced BBB penetration and RONS clearance, which promoted the stroke treatment compared to the free CeO_2_ group [95]. In addition, CeO_2_@ZIF-8 also decreased the activation of microglia and astrocytes after ischemia (Fig. 6e) suggesting its involvement in the immune response, which was consistent with other reports [99, 120–122]. Although CeO_2_@ZIF-8 was relatively large (240 nm) to some extent, it penetrated the BBB successfully due to the ZIF-8 encapsulation. In another study, 8-nm nanoceria was loaded onto poly-(lactide-co-glycolide)-PEG copolymer matrix to form nanocomposites. These composites resulted in an effective decrease in the infarct volume and edema level of the ischemic brain [93].

### Hemorrhagic stroke

Hemorrhagic stroke accounts for ∼20% of all strokes, including two nontraumatic subtypes, intracerebral hemorrhage (ICH) and subarachnoid hemorrhage (SAH) [106]. In the early stage of hemorrhage, brain damage develops with the formation of hematoma and edema, followed by increased intracranial pressure and inflammatory response, inducing secondary brain injury and further neurological deficits [77, 123, 124]. To date, effective treatment that significantly improves the poor outcomes has yet to be discovered. As a regenerative RONS modulator, nanoceria has achieved considerable effects in animal models of hemorrhagic stroke [97–100].

ICH is characterized by intraparenchymal bleeding followed by the development of hematoma, leading to initial mass effect and subsequent inflammation [125]. However, therapies focused on hematoma resolution have achieved little progress, and surgical removal is difficult due to its special location. Perihematomal inflammation sparking secondary brain injury is a potential target for therapeutic intervention [126]. Nanoceria exhibits anti-inflammatory activity *in vitro* [120], as well as *in vivo* ICH models [97, 98]. When PEGylated biocompatible nanoceria was intravenously administered to ICH rats, high accumulation in the hemorrhagic hemisphere was observed, indicating the nanoparticles crossed the damaged BBB. The perihematomal edema caused by ICH was significantly reduced by 68% after treatment with nanoceria, while the hematoma volume was scarcely influenced. In addition, treatment with nanoceria effectively decreased the RONS level and suppressed the recruitment of CD68-positive microglia/macrophages, thus alleviating the inflammation response [97].

**Figure 7.**
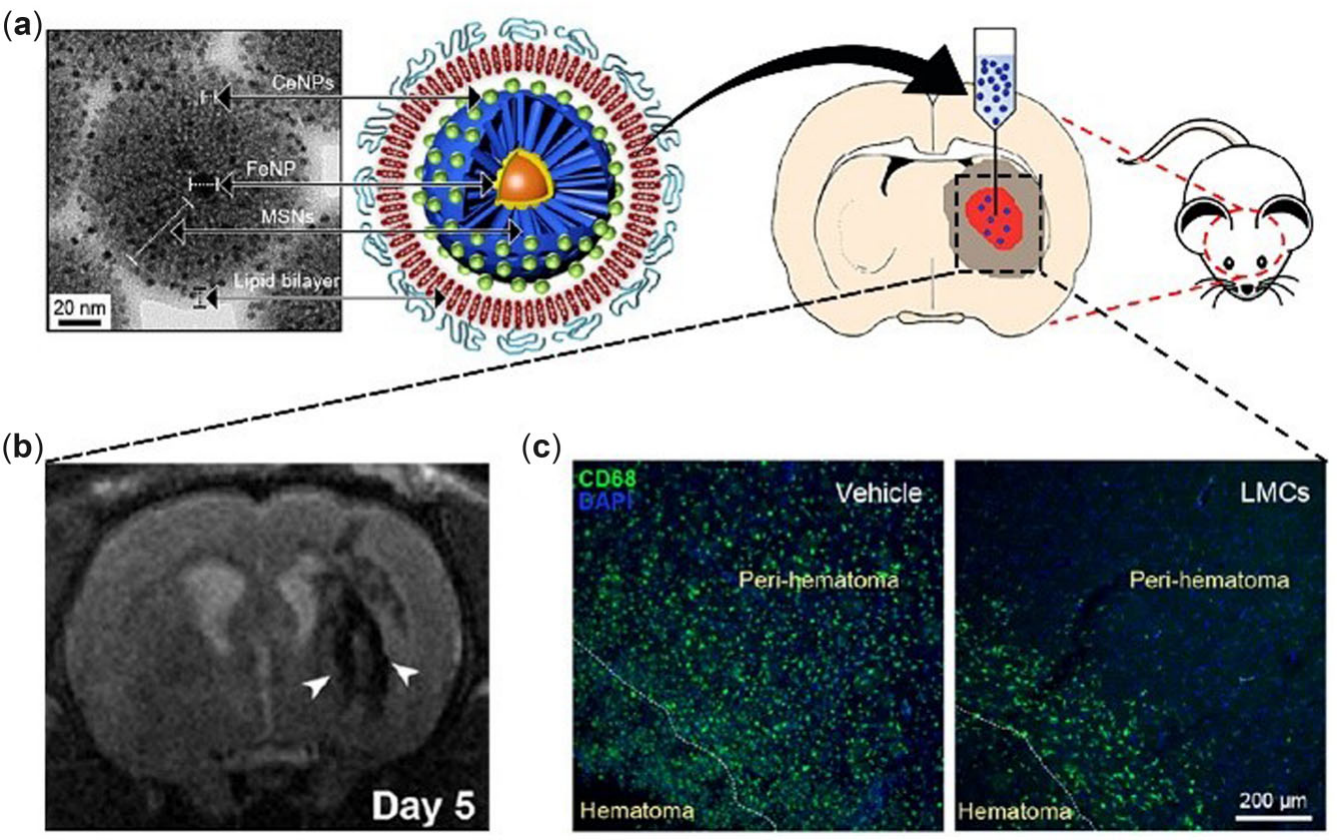
(**a**) Custom synthesized nanocomposite (FeNP@MSNs doped with nanoceria) and its utilization in theragnosis of ICH. (**b**) MRI of ICH brain 5 days after nanocomposite injection. The arrow head points to low signal intensities in the peri-hematomal region, indicating uptake of these nanoparticles by the recruited inflammatory cells. (**c**) Activated microglia/macrophages in vehicle-treated (left) and nanocomposite-treated (right) groups. Adapted with permission from Ref. [98], Springer Nature 2018.

After ICH, resident microglia are activated, and a large number of macrophages migrate to the hemorrhagic area due to chemotaxis, followed by phagocytosis of cellular debris and secretion of proinflammatory cytokines. Moreover, with the activation of the nuclear factor-kappa B signaling pathway, inducible nitric oxide synthase is subsequently expressed, resulting in excessive NO generation [127]. Nanoceria can scavenge excessive NO [72, 120], which acts as a dual mediator of neurotransmission (under normal physiological conditions) and inflammation (under pathological conditions) [128]. In light of the anti-inflammatory capacity, nanoceria was loaded on lipid-coated mesoporous silica nanoparticles for theragnosis of ICH (Fig. 7a). In the synthesis, nanoceria was absorbed by magnetic mesoporous silica nanoparticles (FeNP@MSNs) through electrostatic interactions and exquisitely embedded into the 3.2-nm pores of MSNs. After coating with lipids, FeNP@MSNs doped with nanoceria were intracerebrally injected into ICH rats. Magnetic resonance imaging (MRI) clearly showed that these nanoparticles were successfully recruited to the perihematomal region and spontaneously internalized by microglia/macrophages (Fig. 7b), which was necessary for attenuating the inflammatory response. As expected, treatment with custom synthesized nanoparticles significantly reduced CD68-positive inflammatory cells (Fig. 7c) and brain edema in the perihematomal region after ICH, which was attributed to the antioxidative capacity of nanoceria. In addition to these therapeutic effects, taking advantage of iron oxide nanoparticles, the nanocomposites increased the efficacy of MRI enhancement *in vivo*, which was beneficial for ICH diagnosis [98]. Other examples have been reported utilizing nanoceria for MRI contrast enhancement in disease theragnosis [129, 130].

**Figure 8.**
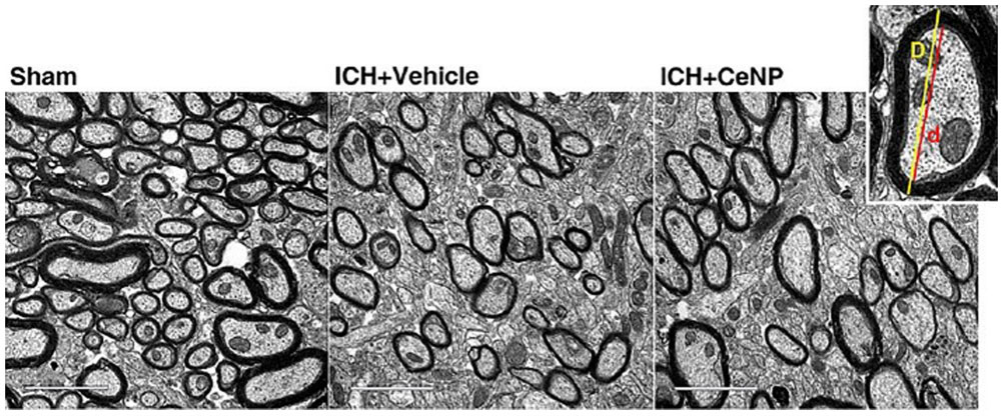
Representative electron micrographs showing myelin sheaths at the perihematomal sites in the striatum after ICH. Adapted with permission from Ref. [99], CC by-NC 4.0, © The Author(s) 2021.

During ICH, RONS accumulation can induce severe damage to white matter, which accounts for at least 50% of the whole human brain volume [131]. A more recent study has reported that PEGylated nanoceria treatment could effectively ameliorate white matter injury and promote the regeneration of myelin sheaths, which surround the axons of neurons and enable the electrical impulses between nerve cells to transmit rapidly. In the ICH group treated with nanoceria, the myelin sheaths at the perihematomal site were thicker than those of the nontreated group (Fig. 8). Namely, PEGylated nanoceria treatment promoted remyelination and improved the integrity of myelinated fibers [99]. In addition, after intraperitoneal injection of nanoceria into a rat model of sciatic nerve crush injury, more myelinated fibers and thicker myelin sheaths were observed [132]. Since crosstalk among oligodendrocytes, microglia and astrocytes is involved in remyelination [133–135], nanoceria may modulate the crosstalk via RONS clearance. After the injury, nanoceria treatment induces a lower expression of M1 microglia and A1 astrocytes and promotes the differentiation of oligodendrocyte progenitor cells, all of which contribute to remyelination and white matter repair. It is consistent with the report that nanoceria might regulate the phenotype of microglia, i.e. from proinflammatory M1 to anti-inflammatory M2 under pathological conditions [121].

SAH accounts for ∼5% of all strokes. Approximately 85% of cases are caused by rupture of an intracranial aneurysm, followed by extravasation of blood into the subarachnoid space. Due to advances in theranostic techniques, the case fatality rates of SAH have markedly decreased during the past several decades. However, many survivors of SAH experience a variety of sequelae, including neurological, cognitive or functional deficits [136]. After blood invades the subarachnoid space, a series of events occur, including a sharp rise in intracranial pressure, a decrease in cerebral blood flow, overproduction of oxyhemoglobin, and other changes in physiology, neurochemistry, molecules and ions. RONS are widely implicated in these damage cascades [137–139].

It was shown that nanoceria synthesized in the aqueous phase and modified with aminocaproic acid/PEG obtained therapeutic effects in the SAH model. *In vitro*, bespoke ceria nanoparticles with high Ce^3+^ concentrations (43–57%) effectively protected macrophages from death when treated with cytotoxic hemin. The blood clot itself could generate RONS via hemoglobin autoxidation, the Fenton reaction of heme and the toxic effects of thrombin. *In vivo*, 1-h after SAH onset, nanoceria was intravenously injected into rats resulting in markedly improved neurological scores and survival rates [100]. It is worth noting that nanoceria might contribute to the aggravation of vasospasm via scavenging NO, which is the endothelium-derived relaxing factor generated from blood vessels [140]. Recently, nanoceria has been shown to induce NO generation from S-nitrosoglutathione (one of the most biologically abundant NO donors), which overturns the conventional concept that nanoceria acts widely as a NO-scavenging agent [141]. Therefore, the interaction between NO and nanoceria under SAH conditions needs to be more clearly elucidated.

### Neurotrauma

Traumatic brain injury (TBI) is the most common cause of death and disability in young people [142]. More than 50 million people experience TBI every year worldwide [143], leading to direct loss of properties and even lives along with indirect impacts on families, friends and society. The primary injury, caused by sudden traumatic damage to the cerebral tissue (vascular breakage and neuronal death), occurs immediately and is almost inevitable. Therefore, therapeutic interventions are mainly focused on the secondary injury, which involves a cascade of biochemical and molecular events, including ionic homeostasis disturbance, release of excitatory neurotransmitters, mitochondrial dysfunction, as well as overproduction of RONS [53].

It was reported that nanoceria improved outcomes after mild TBI. *In vitro*, 10 nM nanoceria added to cell culture 1 h post-injury significantly reduced neuronal death and attenuated glutamate-mediated calcium signaling dysregulation. Furthermore, when applied in a rodent model induced by mild lateral fluid percussion to the rat brain, nanoceria protected the activity of endogenous antioxidants (SOD, catalase and glutathione), thus reducing oxidative damage to the brain. Even though repetitive doses were administered and the most effective dose was the highest dosing paradigm (2.5 mg/kg in total), the distribution of 10-nm nanoparticles in the brain was relatively low [101]. As the mimetic activity of nanoparticles varies with shape [68], ceria nanorods have exhibited better therapeutic effects on alleviation of brain edema than ceria nanospheres after TBI [102].

**Figure 9.**
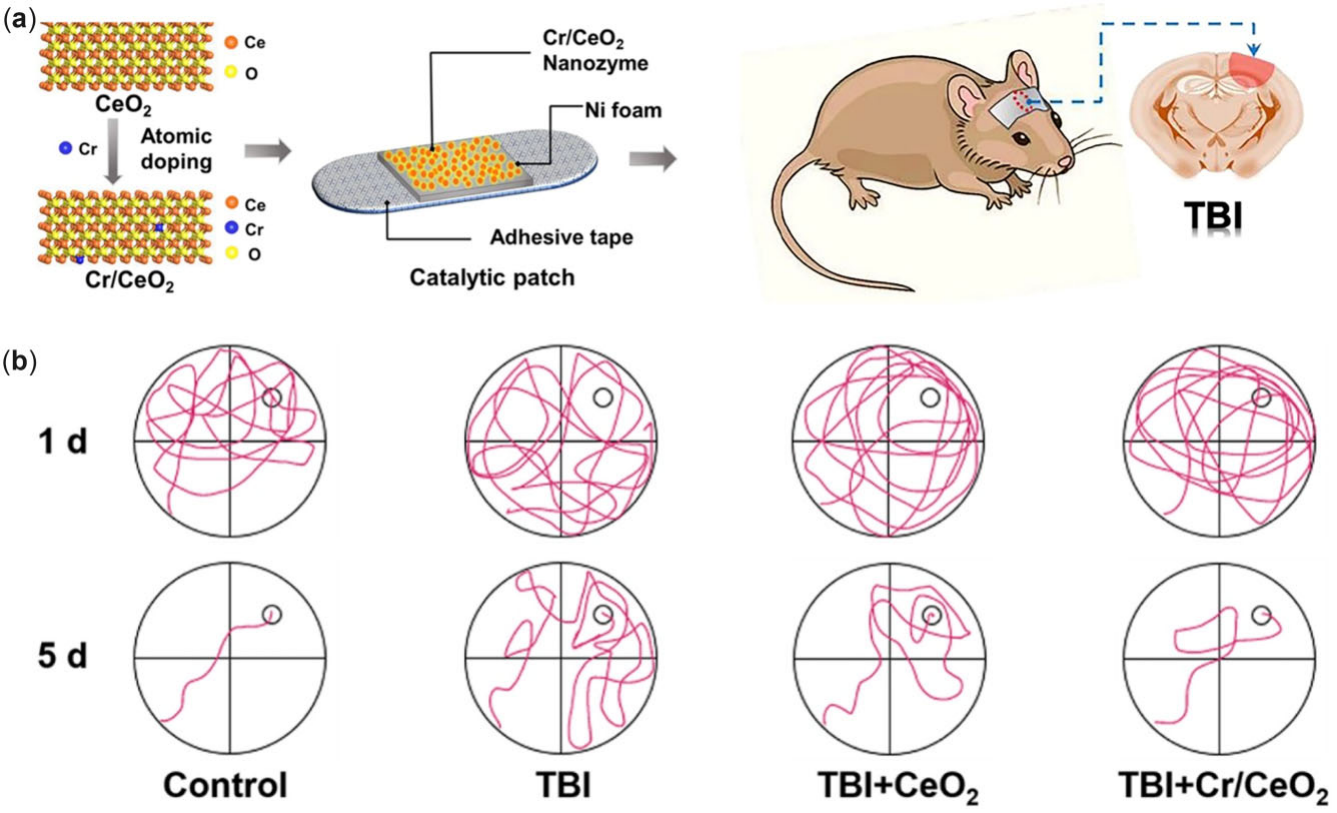
(**a**) Design of non-invasive patch loaded with Cr/CeO_2_ nanozyme for topical treatment in TBI. (**b**) Path of the mice searching platform in Morris water maze test. After 5 days of training, TBI mice with Cr/CeO_2_ nanozyme patch treatment were able to find the platform in a short time, while non-treated TBI mice still could not quickly locate the position of platform. Reprinted from Ref. [104], CC by 4.0, © The Authors 2021.

The conventional delivery route of intravenous injection is likely to generate potential biosafety issues related to the toxicity of nanoparticles, most of which will be transported to other organs through blood circulation. Therefore, it is essential to explore a new solution for TBI treatment to minimize the potential risk. Non-invasive bandage and patch with nanozymes for topical treatment in TBI have been designed [103, 104]. Although traditional antioxidant-based bandages could decrease infection triggered by RONS and inflammation around the wound area, they maintain efficacy for only a short period and need to be frequently renewed, which is inconvenient for brain trauma. To address this issue, a single-atom Pt/CeO_2_-based bandage was constructed by dispersing and trapping single Pt atoms in the CeO_2_ (111) matrix, leading to lattice expansion and catalytic activity enhancement. With the help of Pt single-atom catalyst, the Pt/CeO_2_ system showed several-fold higher activities in RONS clearance than CeO_2_ clusters. In addition, the wound dressing bandage sustained long-term catalytic activity for up to 30 days with little decay. When pasted on the wound of moderate TBI mice for 8 days, it effectively accelerated wound healing to healthy levels while the untreated group only recovered partially [103]. Likewise, the Cr/CeO_2_ nanozyme-based catalytic patch was utilized for TBI treatment as well as shown in Fig. 9. The monodisperse Cr/CeO_2_ nanozyme prepared by the coprecipitation method was loaded onto nickel foams. When doped with Cr^3+^ ions, nanoceria exhibited lattice distortion and a higher Ce^3+^/Ce^4+^ ratio, displaying a several-fold enhancement in enzyme-like activity. In addition, the Cr/CeO_2_ nanozyme at a 10% doping concentration achieved the highest Ce^3+^/Ce^4+^ ratio and the best enzyme-mimetic activity. The patch pasted on TBI mice resulted in excellent wound recovery and improved cognitive function. Furthermore, some of the nanoparticles released from the patch diffused into the brain tissue through the disrupted BBB after TBI, which played a crucial role in modulating RONS and alleviating inflammation [104]. Interestingly, the lattice of nanoceria expanded or contracted when doped with other metal ions because the atomic radius of doping ions is larger or smaller than that of Ce. Through such a doping method, the Ce^3+^/Ce^4+^ ratio of nanoceria could increase, and correspondingly, the enzyme-mimetic activity changes.

**Figure 10.**
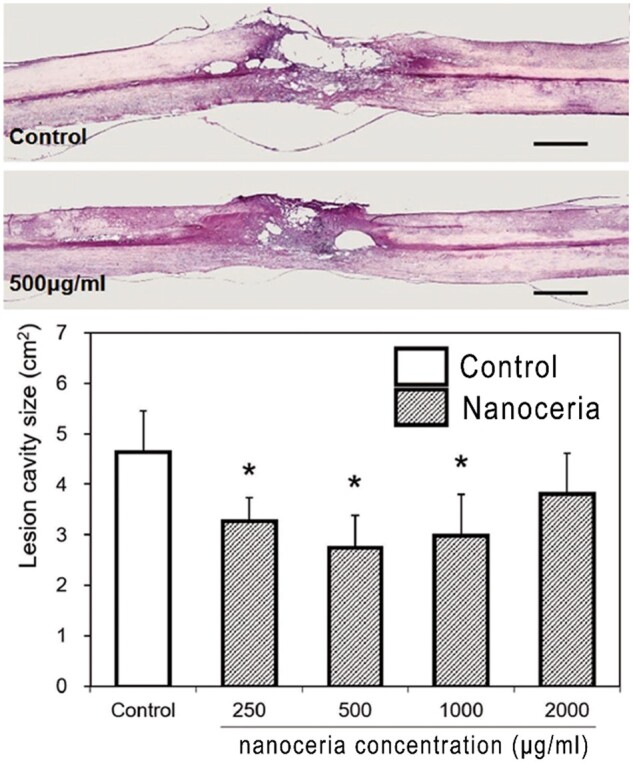
Local injection of nanoceria at concentration of 500 μg/ml significantly reduced the lesion cavity size. Adapted from Ref. [105], CC by 4.0, © The Authors 2021.

Traumatic spinal cord injury, another type of neurotrauma, is an acute injury to the spinal cord caused by external physical impacts similar to TBI [144]. It was initially reported that nanoceria offered neuroprotection to spinal cord neurons isolated from adult rats. Through a microemulsion synthetic process, well-dispersed nanoparticles obtained high biocompatibility and neuroprotection capability *in vitro*. Nanoceria-treated neurons exhibited normal electrical activity and higher survival compared to the control ones [145]. Similarly, nanoceria was capped by plant extracts [146] or loaded onto biopolymer [147, 148] to improve its biocompatible activity. When cocultured with spinal cord cells, these nanocomposites exhibited auto-regenerative and neuroprotective activity, suggesting the therapeutic potential of nanoceria in spinal cord injury treatment. Furthermore, in a rat model of spinal cord contusion, 20-nm nanoceria was locally injected into the lesion cavity 30 min post-injury. Through modulation of RONS, nanoceria treatment alleviated the inflammatory response by downregulating the expression of inducible nitric oxide synthase and proinflammatory cytokines. The cystic cavity size was substantially reduced (Fig. 10), which consequently contributed to locomotor functional recovery [105]. Locally injected nanoceria could be internalized into microglia around the lesion area and modulate their phenotype from proinflammatory M1 to anti-inflammatory M2, as previously reported [121]. A more effective strategy to target microglia and modulate the inflammatory response could be achieved by coating nanoparticles with microglia-specific antibodies. With conjugation of CD11b antibody, ceria–zirconia nanocomposites were intrathecally administered into the spinal canal of mice, resulting in a significantly higher accumulation in microglia other than astrocytes and neurons [149]. Nanoceria may translocate into neurons through axons, which depends on both axonal integrity and electrical activity. In an experiment conducted on frog sciatic nerve fibers, 120-nm nanoceria translocated along the nerve fibers with a speed closer to the slow axonal transport rate [150], which might contribute to the recovery of the damaged nerves [132]. To be mentioned, the bacterial infection commonly occurs after neurotrauma, which seems innocuous and can be treated by antibiotics in most cases. Interestingly, nanoceria has also exhibited antibacterial activity [151, 152], which might promote the recovery of infected neurons and nerves. Normally, CNS is protected from most infections by immune responses and multilayer barriers. Thus, neuron infections mainly occur in the peripheral nervous system and rarely spread into CNS. However, some infections can innervate the peripheral area, resulting in direct or immune-mediated pathology in CNS. With powerful anti-inflammatory activities [120, 121], nanoceria may be effective against neuroinflammation caused by bacterial or viral infection.

## Challenges and future directions

Hereinabove, we have summarized up-to-date applications of nanoceria in acute CNS injury treatment. In general, studies commonly show significant benefits in animal models of CNS injury, which highlights great opportunities that nanoceria and its related biomaterials could be a potential option against oxidative stress in clinical practice. To move this nanomaterial from the bench to the bedside, some challenges remain to be met, leaving future directions for us to follow.

First, the safety problems concerning nanoceria *in vivo* must be considered. Apart from its beneficial RONS-scavenging ability, nanoceria exhibits toxic activities in some cases [153–159]. There are some controversial results regarding the anti- and pro-oxidant activities of nanoceria, which are affected by many factors including particle size, shape, surface chemistry, coatings, local pH and ligands [160]. As these factors greatly influence the biological activity of nanoceria, particles must be well characterized before being delivered into the body, which has been ignored during some works (Table 1).

**Table 1.** Applications of nanoceria in various experimental models of CNS injury

Disease type	Nanoceria size (nm)	Ce^3+^/Ce^4+^ ratio	Coatings	Optimal dosage	Experimental model	Delivery method	Main effects	Date and reference
Ischemia	10 nm	Not mentioned	None	1 µg/ml	Brain slice	Cocultured	30% reduction of ROS in brain slice.	2011 [75]
							Protecting the structure of mitochondria.	
	3 nm	Not mentioned	Phospholipid-PEG	0.5 mg/kg	MCAO	Intravenous	50% reduction of infarct volumes in brain.	2012 [23]
	4.3 nm	34%	Angiopep-2-PEG/edaravone	0.6 mg/kg	MCAO	Intravenous	30% reduction of infarct volumes in brain.	2018 [91]
							Protecting the BBB integrity.	
	Not mentioned	Not mentioned	Biotinylated-LXW7	0.5 mg/kg	MCAO	Intravenous	30% reduction of infarct volumes in brain.	2018 [92]
							Improving neurologic deficit.	
	40 nm	Not mentioned	PEG/PLGA	10 mg/kg	MCAO	Not mentioned	Decreasing infarct volumes and brain edema.	2018 [93]
	2–2.5 nm	Not mentioned	Citric acid/EDTA	60 mg/kg	MCAO	Intraperitoneal	52% reduction of superoxide in hippocampus.	2019 [94]
	20 nm	Not mentioned	ZIF-8	0.4 mg/kg	MCAO	Intravenous	25% reduction of infarct volumes in brain.	2020 [95]
							Decreasing inflammation.	
Hemorrhage	3 nm	43%	Phospholipid-PEG	0.5 mg/kg	ICH	Intravenous	68% reduction of brain edema.	2017 [97]
							Decreasing inflammation.	
	3 nm	Not mentioned	Phospholipid-PEG	10 µg	ICH	Intracerebral	Decreasing microglia/macrophage recruitment and brain edema.	2018 [98]
	3.4 nm	43%	Oleylamine-PEG-DSPE	0.5 mg/kg	ICH	Intravenous	Decreasing inflammation.	2021 [99]
							Promoting remyelination.	
	3 nm	43–57%	Aminocaproic acid-PEG	0.5 mg/kg	SAH	Intravenous	Improving neurologic deficit and survival rates.	2018 [100]
TBI	10 nm	33%	None	0.5 mg/kg	Mild TBI	Intravenous	Reducing calcium dysregulation.	2020 [101]
							Improving cognitive function.	
	3 nm/9 nm	40%/27%	None	11.6 mM	Mild TBI	Retro-orbital	Decreasing cell death and cerebral edema.	2021 [102]
							Improving cognitive function.	
	Not mentioned	Not mentioned	None	Not mentioned	Mild TBI	Non-invasive topical	Decreasing inflammation and wound size.	2019 [103]
							Improving cognitive function.	
	8–12 nm	27%	None	Not mentioned	Mild TBI	Non-invasive topical	Decreasing inflammation and wound size.	2021 [104]
							Improving cognitive function.	
TSCI	19.5 nm	34%	None	10 µg	Spinal cord contusion	Local injected	Decreasing inflammation and cavity size.	2017 [105]
							Improving locomotor function.	
	3–5 nm	Not mentioned	None	10 nM	Spinal cord cell	Cocultured	Neuro-protective effect on the spinal cord neurons.	2007 [145]
	15 nm	Not mentioned	Leaf extract	10 nM	Spinal cord cell	Cocultured	Neuro-protective effect on the spinal cord neurons.	2021 [146]
	15–25 nm	Not mentioned	Chitosan	Not mentioned	Spinal cord cell	Cocultured	Neuro-protective effect on the spinal cord neurons.	2018 [147]
	40 nm	Not mentioned	PCL/RVL	Not mentioned	Spinal cord cell	Cocultured	Neuro-protective effect on the spinal cord neurons.	2020 [148]

PEG, polyethylene glycol; EDTA, ethylene diamine tetraacetic acid; PLGA, poly lactic-co-glycolic acid; ZIF, zeolitic imidazolate framework; DSPE, distearoyl phosphoethanolamine; PCL, poly (e-caprolactone); RVL, resveratrol; MCAO, middle cerebral artery occlusion; ICH, Intracerebral hemorrhage; SAH, subarachnoid hemorrhage; TSCI, traumatic spinal cord injury.

In an experiment exploring the interaction with OH^•^, nanoceria could convert from exhibiting antioxidant to oxidant activity as the concentration of OH^•^ and nanoparticles increased. At high concentrations, more Ce^3+^ ions were introduced into the system, and nanoceria might catalyze the production of OH^•^ similar to Fe^2+^ in a Fenton reaction [161]. When polymer-coated ceria nanoparticles with different surface charges were added to diverse cell lines, they exhibited charge-dependent subcellular localization and cytotoxicity. Nanoceria with a positive or neutral charge entered most of the cell lines, while negatively charged particles internalized mostly in the cancer cell lines. Furthermore, the internalization and localization of nanoceria were closely related to its cytotoxicity. After localized in the lysosomes of these cells, nanoceria exhibited obvious toxicity due to the acidic microenvironment; but little toxicity was shown in the cytoplasm or outside the cells [162]. Based on a large body of literature, nanoceria only displays toxicity in animals when injected at high doses (more than tenths of mg of CeO_2_ per kg of body weight) [163, 164]. For example, rat brain pro-oxidant effects were reported after peripheral administration of 85 mg/kg nanoceria. Without permeating the BBB, 5-nm nanoceria indirectly decreased the ratio of reduced to oxidized glutathione in the hippocampus and cerebellum, which was an indicator of oxidative stress [165]. To date, there is little evidence of toxicity in response to CNS injury treatment with nanoceria *in vivo*, as the administration doses are generally very low, mostly lower than 1 mg/kg (Table 1). ‘When it is not toxic, it is not a medicine’, as the saying goes. Every medicine has side effects, what we should focus on is the safe range of concentrations. Thus, nanoceria is typically safe when injected intravenously at therapeutic doses.

Another issue is regarding how to cross the BBB/BSCB. Many biodistribution studies have unanimously found that the major organs of nanoceria accumulation are the liver and spleen, while a minimal fraction could penetrate the BBB/BSCB into the brain or spinal cord [109, 157, 159]. The most characteristic structure of the BBB/BSCB is the tight junction, an intercellular barrier between endothelial cells [166], which blocks the paracellular passage of most particles. Under normal conditions, the pore size of tight junctions is speculated to be 1.4–1.8 nm; thus, only particles sized ∼1 nm or less can be passively transported through ultrasmall pores [167]. Several methods have been proposed to cross the barrier, including paracellular transport, passive diffusion, cell/carrier-mediated transport and receptor/adsorptive-mediated transcytosis [168, 169]. Specifically, acute CNS injury is usually accompanied by BBB or BSCB disruption, leading to the transient splitting of the junction, which renders a discrete path for nanoparticles. The most conventional solution to facilitate the penetration of larger nanoparticles into the CNS is surface modification. However, the results are not satisfactory in most cases. Another feasible method bypassing the BBB/BSCB is the direct injection of the nanomedicine into the CSF instead of the bloodstream [170]. Through intrathecal administration, nanoparticles sized <10 nm could passively traffic from CSF to the parenchymal tissue [171]. With the help of CSF flow, nanoceria can be dispersed over the whole CNS. In a previous study, PEGylated ceria–zirconia nanocomposites sized 9 nm were intrathecally injected into mice to determine whether the nanoparticles could reach the spinal cord cells. These nanoparticles were widely delivered into the CNS area from the brain to different regions of the spinal cord, and nanoceria was identified in a large part of the spinal cord cells but only in 7% of the brain cells [149]. However, the biodistribution and pharmacokinetics of nanoceria through intrathecal administration have rarely been reported. Considering that these inorganic nanoparticles may be maintained in living bodies for years, long-term experiments are needed to investigate the biochemical changes *in vivo*. Therefore, it is essential to explore these aspects more thoroughly.

How much RONS can each dose of nanoceria scavenge? Furthermore, how much scavenging is too much? To solve these problems, the precise molecular mechanisms by which nanoceria scavenges RONS need to be completely elucidated and understood. Most preclinical studies designed for medical applications of nanoceria are mainly focused on the therapeutic outcome rather than the underlying mechanisms of action. Many results indicate that the enzyme-mimetic activity of nanoceria is closely related to the Ce^3+^/Ce^4+^ ratio, as smaller particles with a larger Ce^3+^ fraction present stronger catalytic effects [172]. However, the Ce^3+^/Ce^4+^ ratio is strongly influenced by the intracellular environment, and what happens to this ratio in the milieu of cells and tissues remains an enigma that has yet to be explored. When internalized into cells, nanoceria was reported to possess a lower Ce^3+^/Ce^4+^ ratio than outside the cells, indicating a net reduction of its oxidation state in the intracellular environment. In addition, a similar ratio was observed in the organelles (cytoplasm, lysosome), which suggested that the net reduction might occur earlier in the process of cellular internalization [173]. Even though nanoceria protects cells against oxidative/nitrosative stress caused by RONS, it seems more rational to treat nanoceria not as a pure antioxidant, but as a mediator of signal transduction partaking in the process of neuronal death and protection. Hence, nanoceria presents somewhat different effects after localized into cells of different types or physiological states [162]. When delivered into the CNS, nanoceria might change the levels of some neurotransmitters, e.g. dopamine, glutamate and NO. It was reported that nanoceria could oxidize dopamine under acidic conditions in aqueous solutions [19] and in human serum [174], while another study reported its promotive effects on dopamine secretion in PC12 neuronal-like cells [175].

In summary, although some work still needs to be accomplished, nanoceria by virtue of its powerful antioxidant activity is considered to be quite promising in the treatment of CNS injury as well as other RONS-related diseases.

## Funding

This work was supported by the Pre-research Project (2020XYY15) of Army Medical University.

*Conflicts of interest statement*. The authors declare that they have no known competing financial interests or personal relationships that could have appeared to influence the work reported in this paper.
